# Erratum

**DOI:** 10.4269/ajtmh.914err

**Published:** 2014-10-01

In *Am J Trop Med Hyg 91:* 138 by Thomas et al, two of the author names were mistakenly combined into one name. The authors are Raymond Césaire and Jacques Rosine. The journal regrets the error.

